# Molecular Imaging of Endometriosis Tissues using Desorption Electrospray Ionization Mass Spectrometry

**DOI:** 10.1038/s41598-019-51853-y

**Published:** 2019-10-30

**Authors:** Clara L. Feider, Spencer Woody, Suzanne Ledet, Jialing Zhang, Katherine Sebastian, Michael T. Breen, Livia S. Eberlin

**Affiliations:** 1 The University of Texas at Austin, Department of Chemistry, 100 E. 24th St, Austin, TX 78712 USA; 2 The University of Texas at Austin, Department of Statistics and Data Science, 2317 Speedway, Austin, TX 78712 USA; 3 Ascension Seton Medical Center, Department of Pathology, 1201W. 38th St., Austin, TX 78705 USA; 4 The University of Texas at Austin Dell Medical School, Department of Internal Medicine, 1601 Trinity St., Austin, TX 78712 USA; 5 The University of Texas at Austin Dell Medical School, Department of Women’s Health, 1301W. 38th St., Austin, TX 7870 USA

**Keywords:** Mass spectrometry, Diagnostic markers

## Abstract

Endometriosis is a pathologic condition affecting approximately 10% of women in their reproductive years. Characterized by abnormal growth of uterine endometrial tissue in other body areas, endometriosis can cause severe abdominal pain and/or infertility. Despite devastating consequences to patients’ quality of life, the causes of endometriosis are not fully understood and validated diagnostic markers for endometriosis have not been identified. Molecular analyses of ectopic and eutopic endometrial tissues could lead to enhanced understanding of the disease. Here, we apply desorption electrospray ionization (DESI) mass spectrometry (MS) imaging to chemically and spatially characterize the molecular profiles of 231 eutopic and ectopic endometrial tissues from 89 endometriosis patients. DESI-MS imaging allowed clear visualization of endometrial glandular and stromal regions within tissue samples. Statistical models built from DESI-MS imaging data allowed classification of endometriosis lesions with overall accuracies of 89.4%, 98.4%, and 98.8% on training, validation, and test sample sets, respectively. Further, molecular markers that are significantly altered in ectopic endometrial tissues when compared to eutopic tissues were identified, including fatty acids and glycerophosphoserines. Our study showcases the value of MS imaging to investigate the molecular composition of endometriosis lesions and pinpoints metabolic markers that may provide new knowledge on disease pathogenesis.

## Introduction

Endometriosis is a debilitating disorder affecting approximately 10% of women within their reproductive years^1,2^. The disease is characterized by the uncontrolled growth of endometrial tissue that lines the uterine walls into other areas of the body, typically within the abdominal cavity^3^. Endometriosis often causes severe and chronic abdominal pain and subfertility, resulting in decreased quality of life for the patient and increased healthcare costs^4^. Despite its prevalence and the detrimental effects this disease has on patients and the healthcare system, endometriosis remains an extremely perplexing disease^5^. Compelling theories on the cause of endometriosis have been proposed, although the biological mechanisms driving endometriosis development are not entirely known, making disease diagnosis and management challenging^6,7^. Further, as diagnostic biomarkers have not been identified and validated, patients are typically diagnosed at the time of laparoscopic surgery, which is the main treatment option for endometriosis patients^8,9^. Endometriosis diagnosis is confirmed by post-operative pathologic evaluation of excised tissue upon visualization of two of the three characteristic histological features: endometrial glands, endometrial stroma, and hemosiderin. Endometrial glands and stroma found in lesions are direct evidence of endometrial tissue outside the uterine cavity and are histologically indistinguishable from the glands and stroma found within healthy uterine endometrium^10^. Conversely, hemosiderin is an iron-storage complex typically observed in tissue following hemorrhage and is associated with various diseases. Thus, hemosiderin is typically considered as a supporting but not a confirmatory histologic feature of endometriosis^11^.

Several studies have suggested that dysregulation of biochemical pathways is a significant factor in endometriosis^12–14^. Chan and coworkers, for example, reported upregulation of sphingolipid metabolism in endometriosis, leading to an accumulation of glucosylceramides and ceramides within the serum and peritoneal fluid of endometriosis patients^13^. Further investigation of molecular alterations of endometriosis tissues could yield new insights into the pathophysiology of the disease while identifying potential biomarkers for preoperative diagnosis and therapy. To this end, mass spectrometry (MS) provides a powerful platform to investigate the molecular composition of biological samples and alterations occurring in a variety of diseases^15–17^. In particular, MS has been used to determine alterations in metabolites, lipids, and proteins in serum, plasma, follicular fluid, urine, peritoneal fluid, and tissue biopsies obtained from endometriosis patients^18–24^. For example, Cordeiro *et al*. recently used electrospray ionization to analyze lipids extracted from follicular fluid of ten endometriosis patients as well as ten healthy controls and determined significant alterations between the lipid profiles of the two groups^18^. Similarly, Li *et al*. applied liquid chromatography (LC) coupled to MS to analyze lipid extracts of eutopic endometrial biopsies from 21 patients with endometriosis and 20 patients with unrelated infertility, and identified 5 potential lipid biomarkers that were capable of identifying endometriosis with 90.5% sensitivity and 75.0% specificity^24^. Nevertheless, the intricacies of endometriosis often complicate identification of biomarkers causally related to the disease. Endometriosis patients often suffer from other diseases such as autoimmune and endocrine disorders, and present increased risk of gynecological cancers^25–27^. This comorbidity typically results in poor sensitivity and specificity of endometriosis biomarkers, making biomarker discovery difficult^28^. Additionally, the heterogeneous histologic nature of endometriosis lesions presents a challenge for traditional MS analysis, as homogenization of tissue specimens prior to MS analysis leads to mixing of lesions with surrounding tissues, potentially affecting the assay specificity for biomarker identification.

MS imaging presents an intriguing approach to study endometriosis tissues. MS imaging enables two dimensional analysis of biological samples, yielding spatially resolved molecular information that can be unambiguously correlated to histologic features^29^. In particular, MS imaging is powerful for the analysis of heterogeneous tissues, as pixel-by-pixel analysis allows segregation of tissue regions, thereby increasing statistical power to investigate discerning molecular features within regions of interest^30^. The most widely used MS imaging technique, matrix assisted laser desorption/ionization (MALDI)^31^, has been successfully implemented towards the analysis of biological tissues and the detection of various diseases. Alternatively, desorption electrospray ionization (DESI) is an ambient ionization MS imaging method in which a spray of charged solvent droplets is directed towards a sample surface to desorb, ionize, and transport molecules to the mass spectrometer for analysis^32^. DESI requires minimal to no sample pretreatment and as a soft ionization technique enables ionization and detection of intact molecular ions^33^. Further, histologically compatible DESI solvent systems allow direct comparison of molecular ion images with histological tissue features on the same tissue section for improved data correlation^34^. Here, we describe a MS imaging study utilizing DESI-MS to spatially and chemically characterize ectopic endometrium from endometriosis lesions and eutopic endometrium prospectively collected from endometriosis patients with the aim of investigating the metabolic differences between eutopic and ectopic endometrial tissues.

## Results

### Molecular imaging of endometriosis lesions and eutopic endometrium

A total of 269 samples from 89 patients undergoing endometriosis resection laparoscopies were collected for our study: 234 endometriosis lesions excised from a variety of locations within the abdominal cavity including the peritoneum, serosal regions of organs such as the rectum, ligaments, ovaries, and fallopian tubes as well as 35 eutopic endometrial tissues collected from a subset of patients undergoing full hysterectomies. The tissues were obtained from patients ranging in ages from 19 to 54 years old. Summaries of the patient demographics are provided in Supplementary Tables S1, S2. No patient exclusions criteria were applied, therefore samples from patients taking hormonal contraceptives to alleviate endometriosis symptoms were included, as well as patients at different time within their menstrual cycles. Of the 234 endometriosis samples, 38 were not analyzed with DESI-MS due to quality control issues such as small sample size (<50 mg) or incorrect gross anatomy tissue diagnosis during surgery.

**Figure 1:**
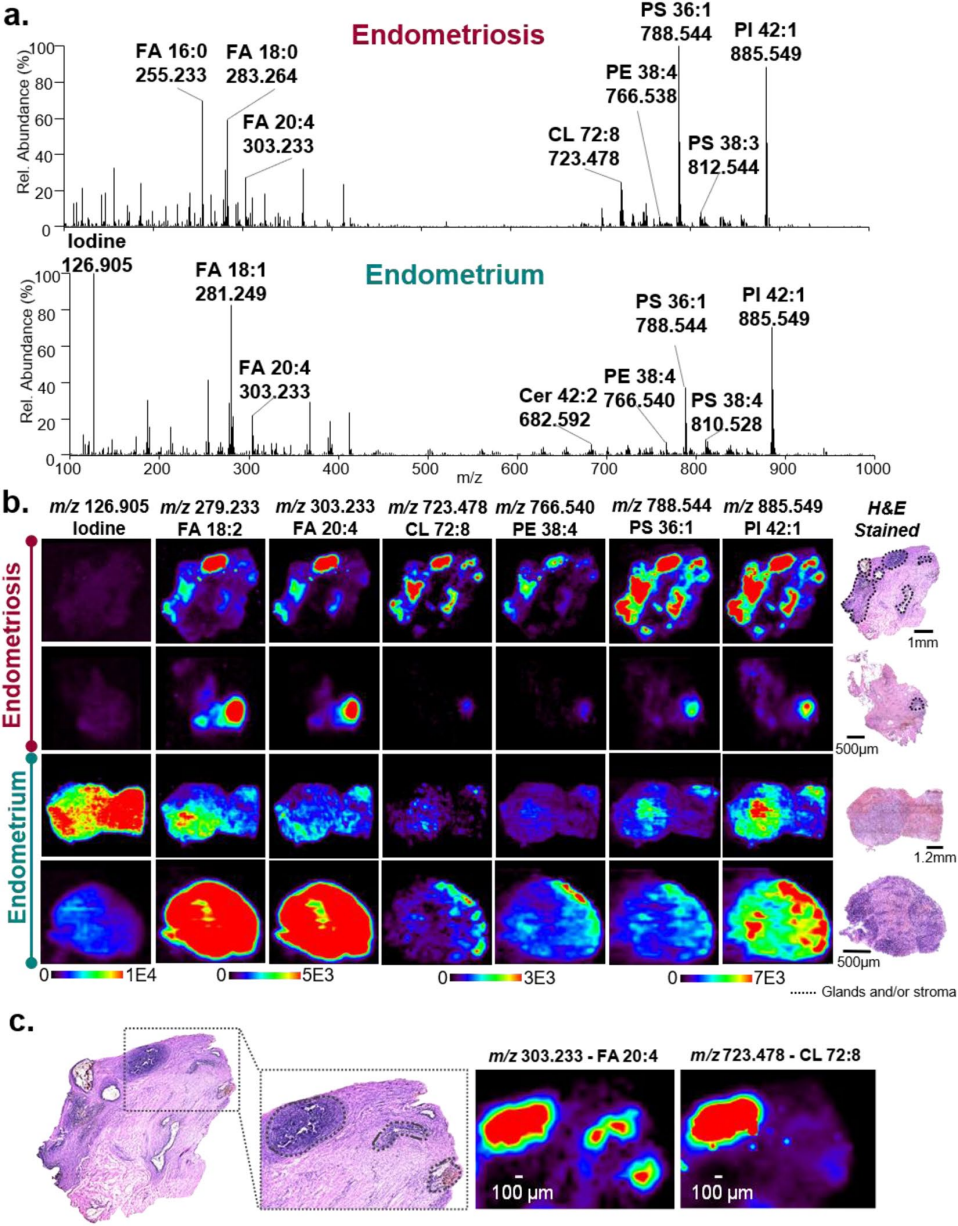
Negative ion mode DESI-MS imaging data acquired from endometriosis and eutopic endometrium tissues obtained from different patients. (**a**) Selected DESI-MS profiles from the endometrial glands within ectopic endometrial tissue collected from endometriosis lesions (top) and eutopic endometrial tissue from inside the uterus (bottom). The spectra shown are an average of 10 scans. (**b**) Selected DESI-MS ion images of endometriosis and endometrium tissues. Regions of endometrial glands and stroma within the lesions are outlined in black on the optical images of the H&E stained tissue sections. (**c**) High magnification view of an endometriosis tissue showing outlined regions of endometrial glands and stroma that spatially correlate to the distributions of various molecular ions detected by DESI-MS imaging, as exemplified by *m/z* 303. 233 and *m/z* 723.478 shown.

Figure 1a shows selected DESI mass spectra of endometrial stroma from an ectopic endometriosis tissue collected from rectum and eutopic endometrial stroma obtained from two different patients. DESI-MS imaging of the tissue sections allowed detection and subsequent identification of a variety of small metabolites and lipids including free fatty acids (FA), sphingolipids such as ceramides (Cer), and glycerophospholipids, such as glycerophophatidic acid (PA), glycerophosphoethanolamine (PE), glycerophosphoglycerol (PG), glycerophosphoinisitol (PI), glycerophosphoserine (PS), and cardiolipin (CL). Note that lipids species are annotated to reflect the number of carbons within their FA chains followed by their level of unsaturation. Example MS/MS data of glycerophospholipids and metabolites is provided in Supplementary Figures S1, S2. As observed in the mass spectra, DESI-MS imaging revealed differences in the relative abundances of a variety of molecular ions between eutopic and ectopic tissues. Within the low mass-to-charge (*m/z*) range, the most notable difference between the two tissue types was the intensity of *m/z* 126.905, tentatively identified as iodide, which was considerably higher in the eutopic endometrial sample when compared to endometriosis lesions. In the higher *m/z* range, the most dominant peak within the mass spectra for the endometriosis lesions is at *m/z* 788.544, identified as PS 18:1_18:0, whereas the most abundant peak in the eutopic endometrium is at *m/z* 885.549, identified as PI 18:0_20:4.

DESI-MS imaging data obtained from eutopic endometrial tissues typically presented a homogenous distribution of molecular ions and thus ion images, reflecting the uniform distribution of endometrial glands and stroma within these tissues. In contrast, DESI-MS imaging of tissues containing endometriosis lesions yielded more heterogeneous images (Fig. 1b). Upon pathological evaluation of the endometriosis tissues, it was determined that areas containing endometrial glands and stroma as small as 50 µm in size were distributed throughout various tissue types including peritoneum and connective stroma. Endometrial glands presented as hollow structures within the tissue sections surrounded by endometrial stroma and therefore exhibited no lipid or metabolite data. Hemosiderin was also observed with high ion signal intensity in a subset of the endometriosis tissue sections analyzed, further contributing to the spatial heterogeneity in the ectopic endometrial tissues. As seen in the selected ion images in Fig. 1b, the most intense ion signal observed from various biological molecules in the DESI-MS ion images typically correlated to tissue regions that contained endometrial glands and stroma, while surrounding connective tissue regions presented low cellular density as well as lower ion signal intensity (Supplementary Figure S3). Figure 1c, for example, shows a higher magnification of an endometriosis tissue obtained from the rectum of a patient, in which high intensities of *m/z* 303.233, identified as FA 20:4, and *m/z* 723.478, identified as CL 72:8, are observed in tissue regions that spatially overlap with endometrial cells (glands and stroma) found within the surrounding serosal connective tissue. There were other tissues and cell types distributed throughout the samples obtained, including inflammatory and ovarian cells, that yielded higher intensities of lipids and metabolites (Supplementary Figure S4). Thus, to assure molecular information from only endometrial cells was being evaluated, we relied on pathological determination of endometrial glands and stroma to precisely locate regions of endometrial cells within endometriosis lesions tissue for comparison to eutopic endometrial tissue. Due to the histological complexity of the tissues and the need to precisely identify regions of pure endometrial cells to extract molecular information, we applied DESI-MS at a spatial resolution of 100 µm. DESI-MS imaging studies using large sample cohorts are often performed at a lower spatial resolution, typically between 150–250 µm^35^. In our study, a spatial resolution of 200 µm was also tested but was not sufficient to clearly define regions of endometrial tissue from the surrounding tissue (Supplementary Figure S5), and thus, 100 µm was used.

**Figure 2:**
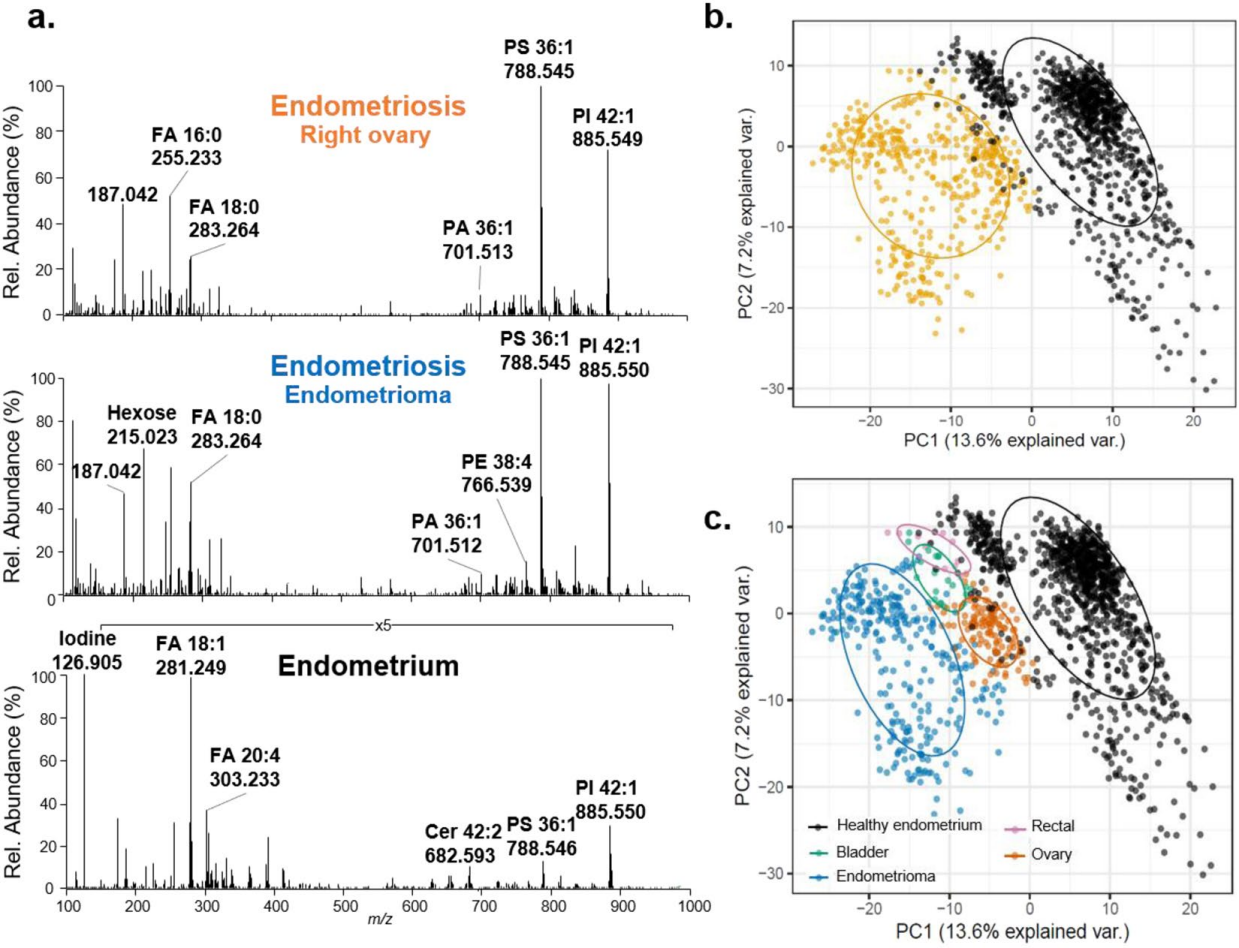
Intra-patient analysis of eutopic endometrium and four endometriosis tissues collected from ovary, rectal, bladder and endometrioma (patient #98). (**a**) Selected DESI mass spectra obtained from three samples, including endometriosis from both the right ovary and an ovarian cyst (endometrioma), and eutopic endometrium. (**b**) PCA score plots of the per-pixel data extracted from all endometriosis tissues (yellow) versus eutopic endometrium tissue (black). (**c**) PCA results of the per-pixel data extracted from endometriosis tissues per region of excision (ovary in orange; rectal in pink, bladder in green, endometrioma in blue) versus eutopic endometrium tissue (black). Ellipses are calculated by a one-sigma ellipse (68% probability) of an estimated bivariate Gaussian distribution for each group.

To evaluate if differences in molecular profiles were potentially related to patient-to-patient variability, eutopic endometrial tissue as well as ectopic endometriosis tissues from various locations within the abdominal cavity were obtained from the same patient and compared. One patient, identified as #98, provided an eutopic endometrial sample as well as four endometriosis tissues collected from the bladder, rectum, left ovary, and an endometrioma. Qualitatively, the trends in molecular profiles detected from eutopic and ectopic endometrial tissues from the same patient appeared to follow similar trends as described for samples from different patients, including an increased abundance of iodide within the eutopic endometrium and higher relative abundance of PS 18:1_18:0 within the endometriosis lesions (Fig. 2a). The molecular patterns detected in endometriosis lesions also appeared to be consistent and independent of the organ from which the sample was collected. To evaluate and visualize intra-patient molecular differences between the eutopic and ectopic endometrial tissues, principal components analysis (PCA) was performed on the data collected for these five samples. As observed in Fig. 2b, the first two principle components allowed for separation between the data obtained from eutopic and ectopic endometrial tissues. Interestingly, separation was also observed within the data obtained for endometriosis lesions collected from different organs. Similar results were observed from tissues collected from different patients (Supplementary Figures S6, S7, S8).

DESI-MS imaging experiments further revealed a unique mass spectrum in specific regions of many endometriosis lesion samples. While this mass spectrum was not correlated to any particular histological feature as determined by histopathologic evaluation, it appeared to co-localize to regions of blood deposits in the tissue. The mass spectra exhibited a unique molecular pattern from any other observed in this study with an unusual high relative abundance of ions that are not typically detected from mammalian tissues (Supplementary Figure S9). Ions detected in the higher mass region *m/z* 950–1050 were identified as n-acylphosphatidylethanolamines (NAPE), rare complex lipids that are formed when the amine group on the PE phospholipid headgroup forms an amide bond with a fatty acid. NAPE lipids were observed within this unique mass spectrum among high abundance of PA, PG, and PI lipids that were also detected throughout the rest of the tissue sample although at lower relative abundances.

### Statistical classification of endometriosis and eutopic endometrium with lasso

**Figure 3:**
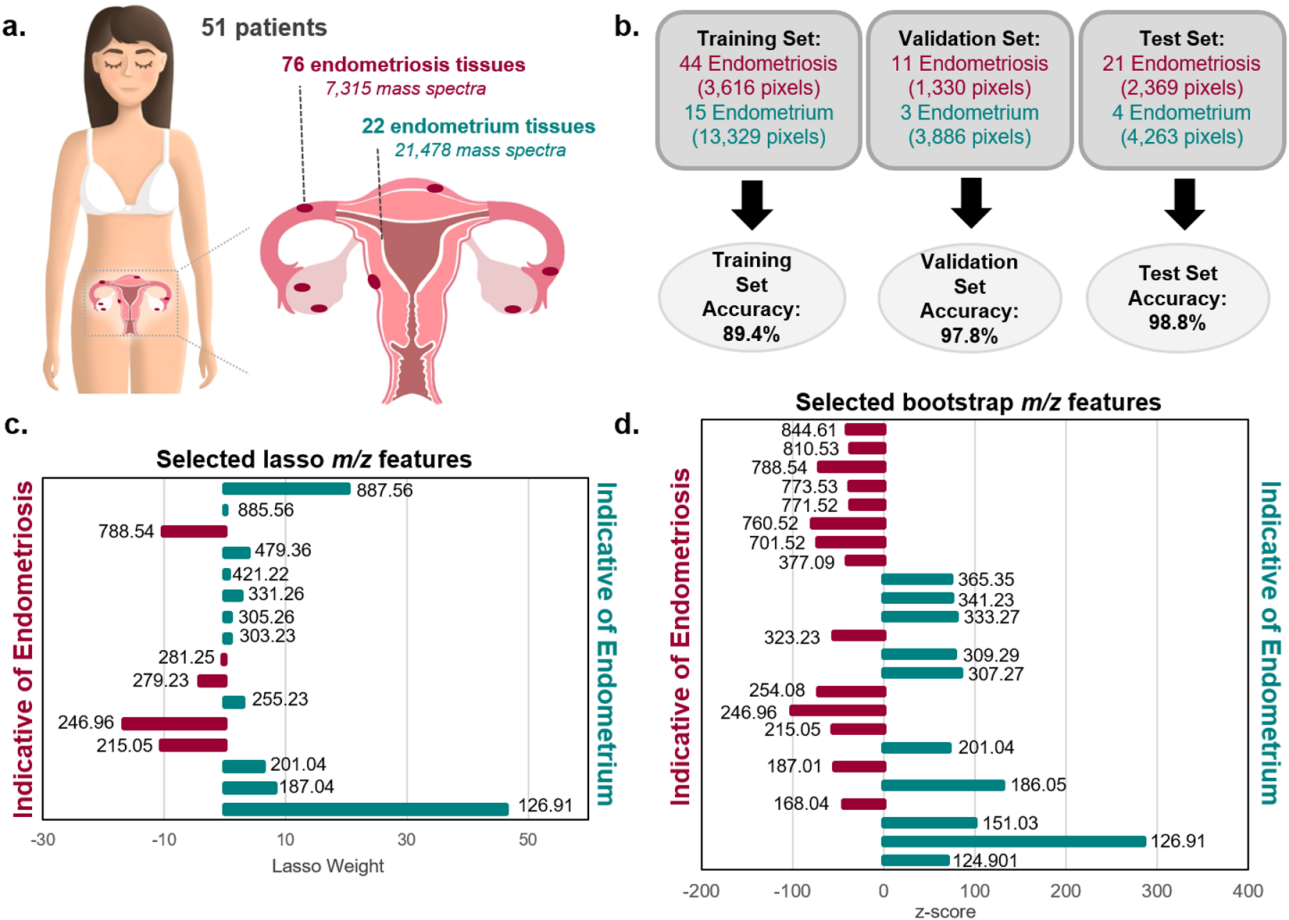
Results of statistical analyses performed on DESI-MS imaging data. (**a**) A total of 98 tissues including 76 tissues with endometriosis lesions and 22 eutopic endometrium tissues were prospectively collected from 51 different patients. Art provided by Viktoriia Tymoshenoko/Shutterstock.com. (**b**) Lasso per-pixel accuracy results for the training, test, and validation sample sets. (**c**) Features selected by lasso as discriminatory for endometriosis and endometrial tissue, where negative weights are more indicative of endometriosis and positive weights are more indicative of endometrial tissue. (**d**) Features selected by bootstrap analysis of the same sample set that are indicative of endometriosis or endometrial tissue, where negative z-scores indicate increase abundance in endometriosis and positive z-scores indicate increased abundance in endometrial tissue.

We next used the lasso statistical method to develop classification models that are predictive of the endometrial stroma from endometriosis and eutopic endometrium, identified by pathological evaluation after DESI-MS imaging. Please note that we analyzed 35 eutopic endometrial samples and 196 endometriosis lesions using DESI-MS imaging. However, upon pathological evaluation of the stained tissue sections after DESI imaging, only 22 of the 35 eutopic endometrial tissue sections and 76 of the 196 ectopic endometriosis samples from 51 patients contained endometrial stroma and were used for statistical analysis (Fig. 3a). A summary of cycle information has been provided for patients whose samples were used for statistics in Supplemental Table S3. The distribution of menstrual cycle information was similar for eutopic endometrial samples and endometriosis samples, and thus we do not believe this factor will have a significant impact on our statistical analysis. Some patients contributed multiple samples including tissues of a variety of endometriosis subtypes as well as eutopic endometrial tissues, as described in detail in Supplemental Table S4. The MS imaging dataset used for statistical analyses was therefore comprised of 28,793 pixels extracted from endometrial glands and stroma within 22 eutopic tissues (21,478 pixels) and 76 ectopic tissues (7,315 pixels). Considering the large number of molecular species detected, we chose to utilize the lasso regularization to construct a logistic regression classifier based on a sparse set of features^36^. The lasso technique has been previously used to create parsimonious and interpretable models with MS imaging data sets that are based on a subset of molecular features that effectively differentiate tissue types^30^. In our study, the samples were split into a training set (n = 59), a validation set (n = 14), and an independent test set (n = 25). The training set was used to train the lasso classification model for distinguishing endometriosis and eutopic endometrium using five-fold cross validation (CV). Optimization of the classifier was performed by optimizing the overall positive percent agreement (PPA), negative percent agreement (NPA), and overall accuracy of the training and validation sets, while maximizing the number of biological features selected.

In the training set evaluation, the lasso model provided a PPA of 96.9% and NPA of 87.3% for diagnosis of ectopic endometrial tissue, yielding an overall accuracy of 89.4% per-pixel. To evaluate possible model overfitting, we then applied the model to a validation set of samples and achieved 93.8% PPA, 99.2% NPA, and an overall agreement of 97.8% per-pixel. Next, we used the model to predict on a withheld, independent test set to further evaluate its robustness. The test set prediction yielded 96.7% PPA, 100% NPA and an overall accuracy of 98.8% per-pixel. Detailed per-pixel and per-sample classification results are provided in Supplementary Table S5, and a summary of the per-pixel results is provided in Fig. 3b. On a per-sample basis, where a majority vote from pixel classification within a sample determines the sample diagnosis, four samples were misclassified: one eutopic endometrial sample from the training set and three ectopic endometriosis samples, one from each of the sample sets. Upon further pathologic review of these samples, the misclassified ectopic endometriosis sample from the test set was determined to be mesothelium rather than endometriosis. Additionally, the misclassified endometriosis sample from the validation set was mixed with hemosiderin tissue. No atypical histologic features were found in the misclassified eutopic endometrial sample or the misclassified endometriosis sample from the training set.

A benefit of the lasso logistic regression method is the selection of predictive features that contribute most heavily to the classification model. The selected endometriosis model consists of 16 tissue specific molecules that allowed for optimal differentiation between these tissue types with varying mathematical weights that signify their contribution to the model (Fig. 3c). Note that a total of 18 features were selected, but 2 of those were determined to be background peaks and thus not biologically relevant. Of the 16 tissue specific features, 5 were selected as indicative of endometriosis while 11 were more indicative of eutopic endometrial tissue. A list of the selected features, their statistical weights in the model, and their tentative identifications are provided in Supplementary Table S6.

### Selection of interesting molecular features by bootstrap empirical Bayes analysis

While the lasso logistic regression performs feature selection during the construction of the predictive model, it does not necessarily select all features within the data set that are significantly different between the two groups if these features do not improve the classification performance. Judging by the out-of-sample predictive accuracy of the final selected lasso model, there appears to be a large amount of signals present to distinguish the two groups. Because lasso favors parsimony, the final selected set of features likely does not fully encompass the molecular differences between the groups.

To more comprehensively evaluate these molecular differences between eutopic and ectopic endometrial tissues, we next performed a nonparametric bootstrap analysis using the same extracted data used for the lasso analysis. Here the goal is to search for features that are marginally different in abundance between the groups rather than to build a parsimonious predictive model. As nearly all features were found to be statistically significantly different in mean intensity between the two groups (Supplementary Figure S10), with *p*-values nearly all approximately zero, we carried out an empirical Bayes analysis to find the most “interesting” features, or those that are outliers when compared to the entire population of features in terms of difference in mean intensities^37^. We hypothesize that each feature is either “uninteresting” or “interesting,” and each of these two groups of features has a corresponding distribution of test statistics, the null and alternative distributions, respectively. Once these distributions are estimated, as illustrated in Supplementary Figure S11, we calculate the posterior probability of each feature being in the “interesting” group of features. We set the target false discovery rate to 20%, yielding the selection of 49 features, 37 of which were determined to be tissue specific (Fig. 3d). A list of tissue specific selected features, along with their *z*-scores, posterior probabilities, and tentative identifications are provided in Supplementary Table S7.

### Molecular differences associated with endometriosis subtypes

Our statistical analyses were performed under the assumption that all endometriosis lesions would have similar molecular patterns. However, endometriosis subtypes characterized by region of excision may contribute to heterogeneity within the collective data, as suggested by our intra-patient analyses. To address the potential for heterogeneity due to lesion subtype, PCA was performed on all the ectopic endometrium. Supplementary Figure S12 shows the PCA results, with different colors representing different tissue subcategories based on their location within the patients. No separation was seen based on endometriosis subcategory, with extensive amount of data overlap. Further, when faceting the PCA score plot to isolate pixels originating from the same patient sample (Supplementary Figure S13), any clustering observed could be attributed to data from the same tissue sample and not subtypes, an observation that was not surprising considering biological variance between patients.

## Discussion

Molecular alterations in the endometrial cells within endometriosis tissues have been suggested as major drivers in the pathogenesis of the disease^13,14,38^. In our study, we applied DESI-MS imaging to evaluate molecular differences between eutopic endometrial tissues and endometrial glands and stroma from endometriosis lesions. Differences in the relative abundances of various metabolite and lipid ions detected by DESI-MS were observed by qualitative comparison of the mass spectra extracted from endometrial cells within eutopic and ectopic endometrial tissues prospectively collected from patients. Notably, the differences observed were consistent in both inter- and intra-patient analyses, and further evidenced by the clustering observed in the PCA performed on the data obtained from five samples from a single patients (Fig. 2 and Supplementary Figures S7, S8), thus suggesting that the molecular changes are associated to biological events rather than patient-to-patient variability such as diet or environmental factors. Statistical analyses of the data obtained from eutopic and ectopic endometrial tissues from 51 patients allowed for clear differentiation of these two tissue types and identification of statistically significant molecular markers, thus indicating occurrence of inherent molecular differences between these tissue types despite the histologically similar nature of the stromal cells (Supplementary Figure S14)^39^.

Lasso and bootstrap empirical Bayes analyses allowed identification of 53 tissue specific features as important in characterizing endometriosis and eutopic endometrial tissue. Note that six features were selected by both the lasso and the bootstrap and are noted in Supplementary Tables S6 and S7. Additionally, the models selected 13 values that were isotopes of already selected molecular ions and were excluded from the final number of features. Therefore, the total number of distinct molecular features selected by both methods not including duplicates or isotopes is 34. Evaluation of the ions selected revealed trends in the molecular information that may give insights into the biological processes occurring in these tissues. Interestingly, fatty acids play a significant role in our models; shorter acyl chain FA (FA 18:1 and FA 18:2) were more characteristic of endometriosis tissue while longer fatty acid chains with higher levels of unsaturation (FA 20:2, FA 20:3, FA 20:4, FA 22:3, and FA 22:4) were more characteristic of eutopic endometrium. Previous studies have evaluated the effect of polyunsaturated FA, and more specifically omega-3 FA, on the survival of endometrial cells in culture and determined that the presence of certain highly saturated fatty acids suppressed endometrial cell survival. Similarly, serum from women with endometriosis was found to have lower levels of the same polyunsaturated FA, eicosapentaenoic acid (FA 20:5), than the serum of healthy controls^40^. While FA 20:5 was detected at very low relative abundance at *m/z* 301.217 by DESI-MS imaging, a number of polyunsaturated FA with the same acyl chain length were selected as indicative of eutopic endometrium. While we are currently unable to determine if the polyunsaturated FA within our data are omega-3 fatty acids due to inability of traditional MS fragmentation methods to determine double bond position, this strengthens the hypothesis that highly unsaturated FA may be at decreased levels in endometriosis lesions.

Studies have suggested that molecular events related to dysregulated phospholipid and sphingolipid metabolism play a role in endometriosis pathogenesis^13,18,22,23,41–43^. In particular, glycerophosphocholine (PC) species have been detected at altered levels in biological specimens from endometriosis patients when compared to healthy controls^22,41–43^. For example, Vouk *et al*. found the upregulation of 5 PC lipids in the serum of patients with endometriosis but later found 4 different PC species downregulated in the peritoneal fluid of endometriosis patients compared to controls. Most of these studies were performed using MS in the positive ion mode, in which zwitterionic PC, sphingomyelins, and PE lipids are more easily detected, while our study was performed in the negative ion mode, which favors detection of phospholipids with negatively charged headgroups. A previous study reported higher abundance of a potassium adducted PS species within the follicular fluid of control patients compared to patients with endometriosis^18^. In contrast, multiple PS species including PS 16:0_18:1, PS 18:1_18:0, PS 18:0_20:4, and PS 18:1_22:0 were selected as indicative of endometriosis tissue rather than eutopic endometrium in our statistical models. PS are typically confined to the inner membrane of eukaryotic cells, but are known to be involved in multiple cellular processes including homeostasis and programmed cell death^44^. The upregulation of PS in endometriosis lesions could be a result of increased blood coagulation of the endometrial tissue during or after a woman’s menstrual cycle, when the endometrial lining within the uterus is expelled from the body due to a decrease in estrogen levels. Endometriosis tissues respond similarly to hormonal levels but are trapped within the adhesions, which could potentially signal coagulation cascades, triggering exposure of PS on the outer surface of blood platelets^45^. In addition to PS, multiple PA and PG species were selected by the bootstrap empirical Bayes analysis as having increased relative abundance within the ectopic endometriosis tissue while PI 18:1_20:4 was selected as a feature more indicative of eutopic endometrium.

A variety of small metabolites were also selected as features within our models for endometriosis and eutopic endometrium. For example, hexose, presumed to be glucose due to its abundance within tissue, was selected in its chlorine adducted form as a feature indicative of endometriosis lesions in both models. Previous studies have shown alterations in the glucose transporter proteins in the eutopic endometrium of women with endometriosis and their matching endometriosis lesions, likely associated to different glucose uptake between these cell types^46^. As expected from qualitative evaluation of the mass spectra, iodine was also selected as a feature indicative of eutopic endometrium in both our statistical analyses. The presence of this ion within the eutopic endometrium may be related to the proximity of this tissue to the cervix which is known to contain a high concentration of iodine when compared to other body areas^47^. Additionally, some research studies also suggest that endometriosis is an iodine-deficiency disease and thus would account for the lower levels of iodine within the ectopic endometrium detected in our study^48^. An ion at *m/z* 246.951 was also selected by both the lasso and the nonparametric bootstrap models as indicative of endometriosis. Interestingly, an ion at *m/z* 246.951 has been previously visualized in other DESI-MS imaging studies and suggested a potential indicator of normal tissue when compared to squamous cell carcinoma and lung adenocarcinoma tissues^49^. Based on tandem MS (Supplementary Figure S15) and exact mass measurements, we identified *m/z* 246.951 as a heavily fluorinate compound with the molecular formula C_3_HF_6_SO_4_. Due to the fluorinated nature of this compound, we hypothesize that this ion corresponds to a drug metabolite from the breakdown of the surgical anesthetic commonly used during endometriosis surgery, rather than an endogenous molecule. Its presence within our statistical models, while undesirable, is not unexpected considering the untargeted nature of MS imaging analyses, and emphasizes the importance of retroactive investigation of the molecules selected by the statistical models using these data sets. Due to its exogenous nature, we have removed this feature from the list of tissue-specific features shown in Supplementary Tables 5 and 6.

Interestingly, DESI-MS imaging experiments revealed a unique mass spectrum characterized by high relative abundance of NAPEs in specific regions of many endometriosis lesions samples. NAPEs constitute about 0.01% of mammalian cell membranes and are often correlated to cellular stress conditions such as ischemia and Parkinson’s disease^50^. Previously, NAPE lipids were detected by DESI-MS imaging in rat brains tissues that had been subjected to focal brain ischemia, showing the slow increase of these lipids in the ischemic area within 24 hr of the stroke, suggesting that these species accumulated during cell death^51^. In our study, these unique molecular patterns were observed in areas presenting cautery damage incurred during the endometriosis surgery. We thus hypothesize that the cellular damage endured through the electrocautery process induced cell death and caused accumulation of NAPEs and other phospholipid species within the cauterized tissue regions. The localization of these unique mass spectra to areas consisting of blood deposits also suggests the cells affected may be related to the vascular system. While very interesting, the accumulation of NAPE species detected are likely not causally correlated to the presence or absence of endometriosis lesions but rather an external factor and thus were not considered in our statistical analysis, although further investigation is needed.

PCA analysis of the data obtained from endometriosis lesions was performed to evaluate if the molecular information obtained correlated with disease subtypes (Supplementary Figure S12). Endometriosis lesions may be separated into three categories: ovarian, peritoneal, and deep infiltrating endometriosis, depending on where the lesion was located within the body^52^. Researchers have suggested that these categories should be treated as separate entities with potentially different causes, symptoms, and treatments^28^. In our study, while some clustering was observed within the data obtained from four endometriosis lesions collected from the same patient (Fig. 2c), PCA analysis of all the DESI-MS imaging data obtained from endometriosis lesions across patients did not show significant separation or clustering based on location of excision. These results suggest that the molecular data acquired by DESI-MS imaging in this study does not allow for subtyping based on tissue location across different patients. This observation is also partially supported by the high predictive accuracy achieved by our lasso model in which the data were collectively combined into a single endometriosis class.

DESI-MS imaging was used to investigate the spatial distribution and metabolic profiles of the eutopic endometrium and endometrial stroma from endometriosis lesions. While greatly less metabolic coverage is achieved with DESI-MS imaging than traditional MS techniques such as LCMS, the spatial resolution provided by DESI-MS imaging (100 µm) allowed collection of spatially-resolved metabolic information and showcase the usefulness of MS imaging in segregating endometriosis lesions within complex tissues. Despite being histologically similar, DESI-MS imaging and statistical analyses allowed identification of distinct chemical differences between the two tissue types, which could be indicative of biological changes associated with differences between eutopic endometrium and endometriosis lesions. While the biological processes that may be driving these differences are unclear, our results suggest that metabolic differences between these two tissue types exist which could potentially be translated to future discoveries of clinical biomarkers of endometriosis. However, it remains a possibility that these differences between eutopic and ectopic endometrium are due to their immediate environment rather than inherent differences between eutopic and ectopic endometrial tissues. Further, as patients on hormonal treatment were included in our study to maximize sample accrual, metabolic alterations due to hormonal treatment, as previously observed in plasma and serum^53,54^, could have potentially affected the results obtained in our analyses. Although inclusion of tissues from patients on hormonal treatment is a limitation of our study, statistical analysis of the data obtained from tissues of patients both on and off hormonal treatment for endometriosis should allow selection of metabolic markers that occur within both groups, independent of hormonal treatment. Further studies are planned to evaluate metabolic changes in tissues from endometriosis patients depending on hormonal treatment. Additionally, our study does not determine if the eutopic endometrium from endometriosis patients is molecularly similar to eutopic endometrium from healthy women, as these samples were not available for our study. Future studies to validate and elaborate these findings, including integration of healthy controls, symptom correlation to specific molecular features, and evaluation of other semi-invasive or non-invasive methods for endometriosis detection are currently being pursued.

## Methods

### Tissue sample collection

Human endometriosis lesions (n = 234) and eutopic endometrial tissue (n = 35) were prospectively collected from 89 patients undergoing both conservative and radical endometriosis surgeries by Dr. Michael T. Breen at Dell Medical School. Tissues were collected under approved IRB protocols from both the University of Texas IRB and the Seton Family of Hospitals IRB. Informed consent was obtained from all patients participating in this study. All endometriosis lesions samples were excised using unipolar electrical scissors during a laparoscopic procedure, while eutopic endometrium samples were collected only from patients undergoing full hysterectomy using a standard scalpel after the completion of the procedure. Samples were stored at 4 °C in airtight containers atop moist gauze until they could be flash frozen in liquid nitrogen, typically within 6 hours of excision. Samples were then stored in a freezer until sectioned. Tissue samples were sectioned at 16 µm thick sections using a CryoStar NX50 cryostat (Thermo Scientific, Waltham, MA) and thaw mounted onto glass microscope slides. After sectioning, the glass slides were stored in a −80 °C freezer. Prior to MS imaging, the glass slides were dried in a desiccator for ~15 min.

### Chemicals

The acetonitrile and dimethylformamide used for the DESI-MS spray solvent were purchased from Fisher Scientific (Waltham, MA) and Sigma Aldrich (St. Louis, MO), respectively. All solvents used for histological staining, including methanol, hematoxylin, bluing reagent, eosin y, ethanol, and xylenes were purchased from Fisher Scientific (Waltham, MA).

### DESI-MS imaging

All experiments performed were carried out in accordance with the approved IRB protocol. A DESI 2D system (Prosolia Inc., Indianapolis, IN) was used for tissue imaging at a pixel size of 100 µm. DESI-MS imaging was performed in the negative ion mode from *m/z* 100–1500, using a hybrid LTQ-Orbitrap Elite mass spectrometer (Thermo Scientific, San Jose, CA) at 60,000 resolving power (at *m/z* 200) using dimethylformamide:acetonitrile 1:3 (v/v) at a flow rate of 1.4 µL/min unless otherwise stated. The approximate time-per-scan was 1.24 seconds with a stage speed of 94.3 μm/s. The mass accuracy obtained was <10 ppm. All DESI-MS spectra shown throughout the manuscript are an average of approximately 5 scans. Ion images were assembled using Biomap (Novartis) software. The ion images were made using the absolute ion intensity counts from the spectra, with 6000 counts corresponding to 100% relative abundance. The ion images were smoothed using the interpolate function within BioMap. All DESI-MS imaging data is available on METASPACE^55^.

### Histopathology and light microscopy

The same tissue sections analyzed by DESI-MS imaging were stained using standard hemotoxilyn and eosin (H&E) staining protocol. Pathologic evaluation was performed by Dr. Suzanne Ledet. Regions of definite and probable endometrial stroma, endometrial glands, and hemosiderin were located within the endometriosis lesions. Eutopic endometrial tissue was confirmed by pathology and regions containing pure endometrial tissue were noted. Light microscopy images of the H&E stained slides were taken using the EVOS FL Auto Cell Imaging System (Invitrogen, Thermo Fisher Scientific, Waltham, Massachusetts, USA).

### Identification of molecular ions

Metabolite and lipid species were identified using high mass accuracy measurements and collision induced dissociation (CID) and high-energy collision induced dissociation (HCD) tandem MS analyses, performed using the Orbitrap as the mass analyzer of the LTQ-Orbitrap Elite mass spectrometer. Fragmentation was performed by rastering the DESI source over serial tissue sections at an increased flow rate (3 μl/min). The isolation window used was 1 *m/z* with an energy ranging from 10–30 normalized collision energy for CID and 40–90 for HCD. Fragmentation patterns were compared to literature reports and compared to data from Lipidmaps (www.lipidmaps.org) and Human Metabolome Databases (www.hmbd.ca). Lipids were characterized by fatty acid composition, but the stereochemistry of the chains and double bond position is unknown.

### Statistical analysis

A total of 22 of the 35 eutopic endometrial tissue sections analyzed and 76 of the 196 ectopic endometriosis tissue sections analyzed from a total of 51 patients (Supplementary Table S4) contained endometrial stroma and thus could be used for statistical analysis. Lasso statistical analysis of the DESI-MS imaging data was performed to build a classification method capable of distinguishing between eutopic and ectopic endometrial tissue, identify biological features of greatest importance within this classification model, and evaluate if the molecular alterations of endometriosis are independent of lesion location. MS imaging data was converted to an mzML format using MS Convert (version 3.9.11748) and imzML format using imzMLConverter (version 1.3) opensource software programs without the addition of any data filters^56,57^. DESI-MS imaging data from pure histological regions of endometrial glands and stroma from endometriosis lesions and endometrial tissue were extracted using MSiReader software^58^. The *m/z* range was discretized by performing hierarchical clustering and cutting the resulting dendrogram at distance 0.05. Peaks appearing in more than 10% of the pixels were retained for analysis, and intensities for each spectrum were normalized by their median non-zero intensity. The samples were randomly divided into training, validation, and test sets of samples. A logistic regression model was constructed using lasso regularization^36^ with the glmnet package in the R programming language^59^, and the lasso complexity penalty was selected using five-fold CV. The first penalization parameter selected yielded a model containing 6 background ions, potentially due to batch effects incurred during the analysis. To alleviate this, we considered models using a larger complexity penalization parameter resulting in fewer selected features, even if their performance in CV was lower than the original selected model. The final model was selected to optimize the overall PPA, NPA, and accuracy of the training and validation sets, while minimizing the ratio of background ion to tissue-specific features. To evaluate the performance of the final selected model, PPA, NPA, and overall agreement of the test set is reported. In our study, PPA describes the true positive rate for the identification of ectopic endometrial tissue while NPA describes the true negative rate.

The same preprocessing methods used for the lasso statistical analysis were also used for the bootstrap empirical Bayes analysis, including hierarchical clustering, peak filtering, and normalization. After preprocessing, we calculated the log-ratio of mean intensities between the two groups. Then, we used the bootstrap method to estimate standard errors for these log-ratios. Specifically, for one bootstrap sample of the log-ratio of mean intensities for one feature, we resampled with replacement the intensities for that feature from all pixels, retaining the group label. The *z*-values were calculated as the observed log-ratio of mean intensities, divided by their standard error. The observed test statistics and their standard errors are shown in Supplementary Figure S10, and it is clear that the vast majority of features are statistically significantly different from zero, when considering the traditional sharp null hypothesis of no difference in mean intensity between the two groups of pixels. Therefore, we then carried out an empirical Bayes analysis to detect “interesting” features^37^. Briefly, this approach assume a two-groups model for observed test statistics, that features are either “interesting” or “uninteresting,” and that both groups of features have a corresponding distribution, called the alternative and null distributions, respectively. This deviates from the usual approach of sharp hypothesis tests of no difference in expression level. We estimate the null distribution empirically, assuming it is normally distributed, and we use moment matching from the middle two quartiles of test statistics to estimate the mean and variance of this normal distribution. The full mixture density is estimated using a spline interpolation of a histogram of all the test statistics (Supplementary Figure S11). The prior probability of features being “uninteresting” is estimated using these two estimated densities. From these components we calculate the local FDR, or posterior probability of being “uninteresting,” for each feature, and report features for which the local FDR is less than 20%.

Principal component analysis (PCA) was used to evaluate quantitative differences in the data owing to subtype. Intensities for each mass spectra were normalized by the median-log non-zero intensity to account for higher intensity peaks showing higher variance, and thus poorly representing the overall variance in the spectra^60^.

## Supplementary information

Supplementary Information
