# Supplementary material for: Molecular Imaging of Endometriosis Tissues using Desorption Electrospray Ionization Mass Spectrometry
Source: Sci Rep. 2019 Oct 30;9:15690. doi: 10.1038/s41598-019-51853-y (PMC6821845; doi:10.1038/s41598-019-51853-y)
Supplement: Supplementary file 1 — Supplementary Information [file 41598_2019_51853_MOESM1_ESM.docx]

Supplementary Information

**Molecular Imaging of Endometriosis Tissues using Desorption Electrospray Ionization Mass Spectrometry**

Clara L. Feider^a^, Spencer Woody^b^, Suzanne Ledet^c^, Jialing Zhang^a^, Katherine Sebastian^d^, Michael T. Breen^e^, Livia S. Eberlin^a^

^a^The University of Texas at Austin, Department of Chemistry, ^b^The University of Texas at Austin, Department of Statistics and Data Science, ^c^Ascension Seton Medical Center, Department of Pathology, ^d^The University of Texas at Austin Dell Medical School, Department of Internal Medicine, 1601 Trinity St., Austin TX 78712 ^e^The University of Texas at Austin Dell Medical School, Department of Women’s Health, 1301 W. 38^th^ St., Austin TX 7870

**This PDF file includes:**

Supplementary Figures S1 to S15

Supplementary Tables S1 to S7


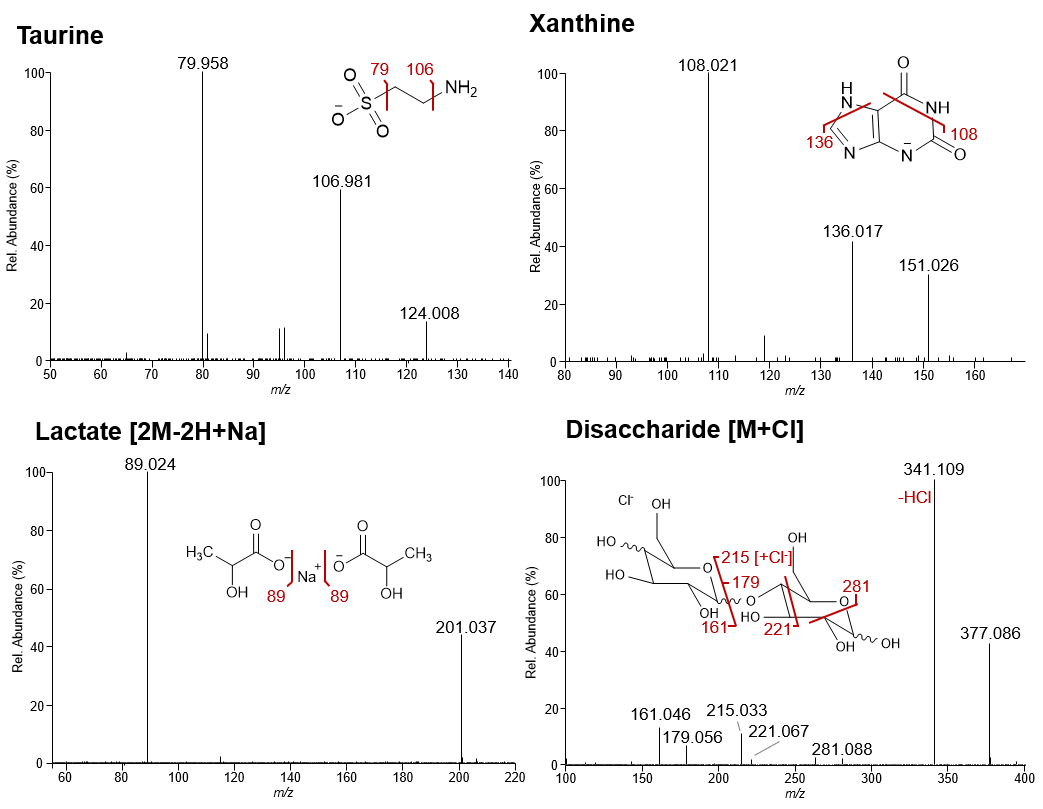


**Figure S1.** Example MS/MS data collected to identify metabolite species detected during DESI-MS imaging experiments of endometrium and endometriosis lesions that were later selected as features within either lasso or empirical bootstrap analyses.


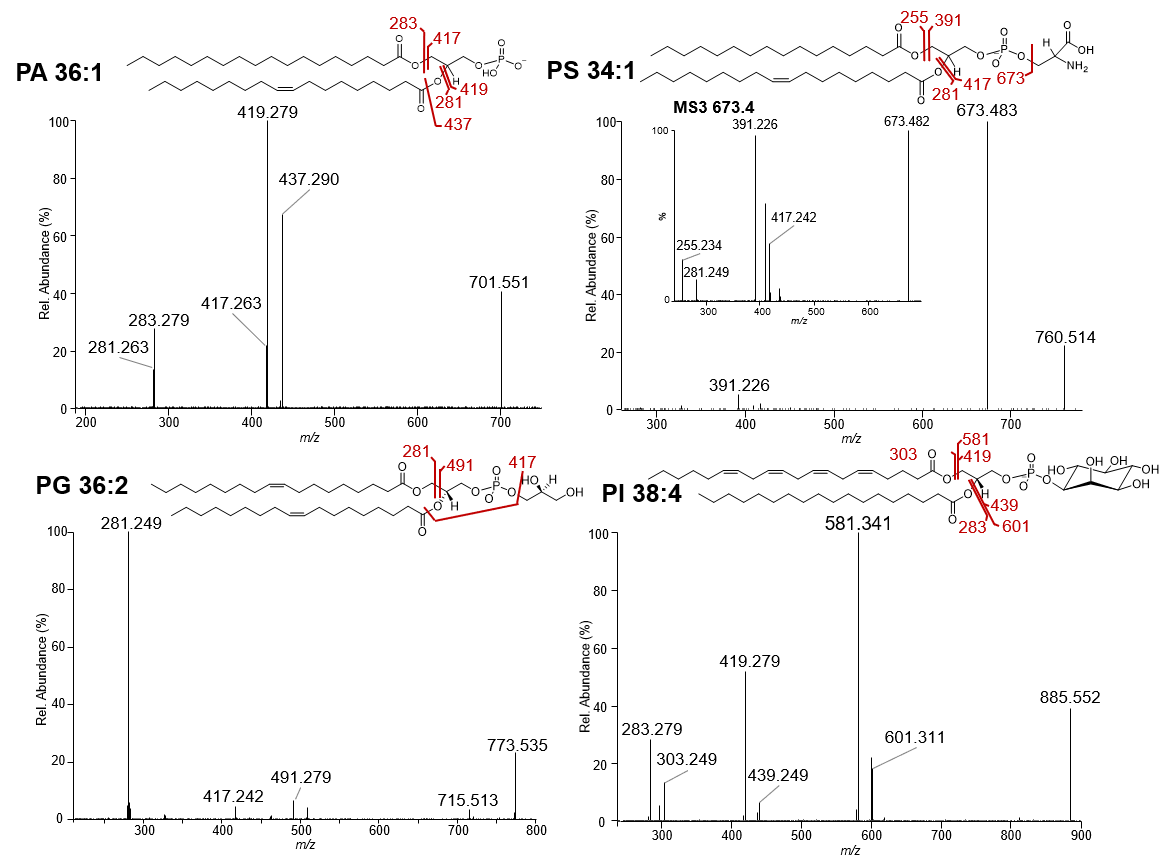


**Figure S2.** Example MS/MS data collected to identify glycerophospholipid species detected during DESI-MS imaging experiments of endometrium and endometriosis lesions that were later selected as features within either lasso or empirical bootstrap analyses.


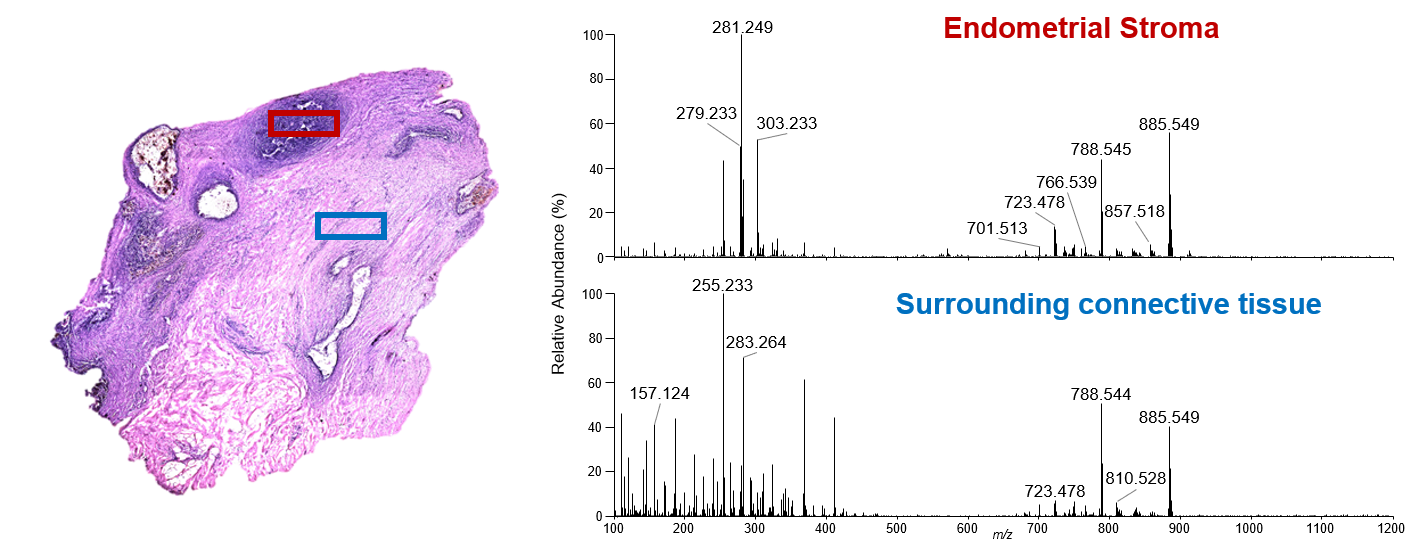


**Figure S3.** DESI-MS imaging mass spectra from endometrial stroma region (Top) and surrounding connective tissue (Bottom). The colored boxes on the optical image (Left) correspond to the endometrial stroma (Red) and the connective tissue (Blue) from which 5 spectra were averaged to display. The relative abundance for the endometrial stroma is about one order of magnitude more intense (NL: 3.76E4) compared to the connective tissue (NL: 4.86E3), hence the lower overall signal intensity exhibited throughout the ion images shown in the manuscript.


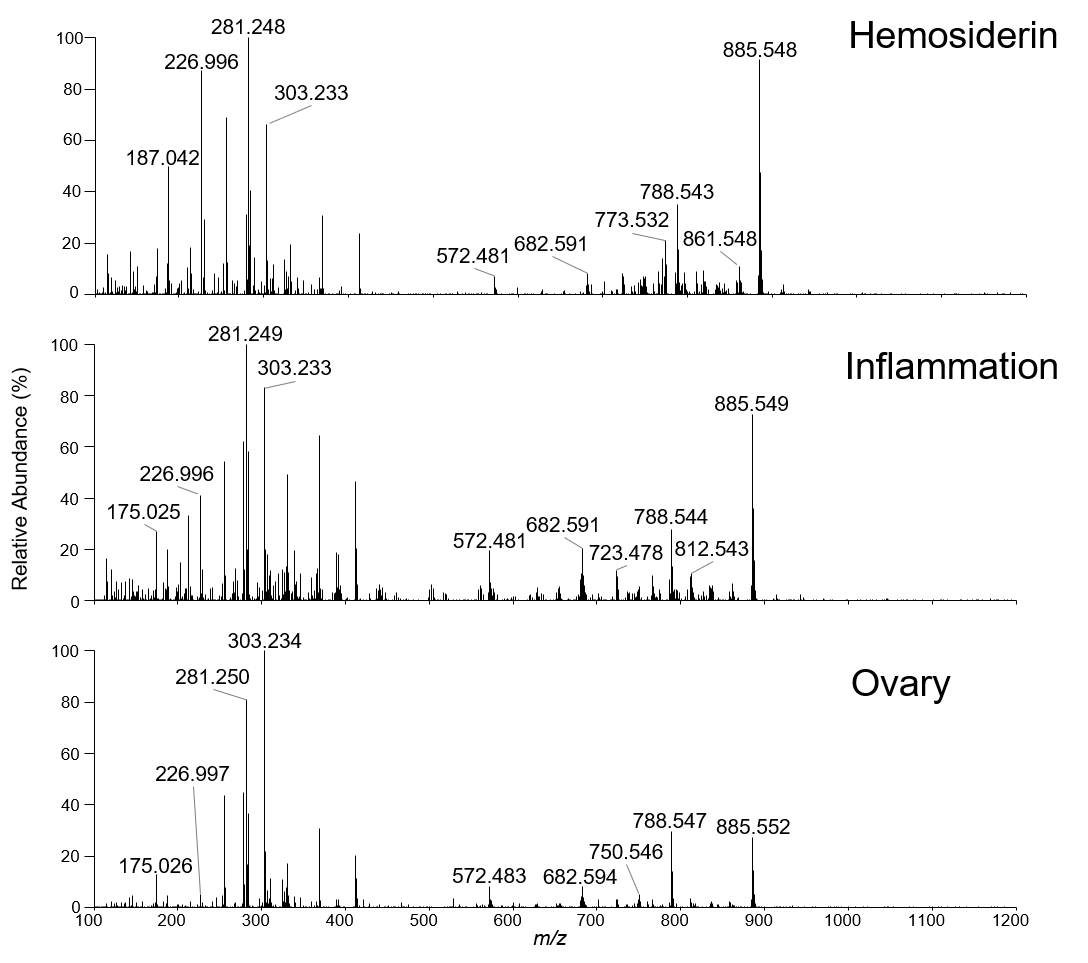


**Figure S4.** DESI-MS imaging mass spectra from other regions of tissue involved in this study, including hemosiderin (Top), inflammatory cells (Middle), and ovary (Bottom).

**Figure S5.** Comparison of 2D DESI-MS ion images of *m/z* 885.549 of an endometriosis lesion tissue section at 200 µm and 100 µm spatial resolution. The solvent used for the 200 µm imaging was 1:1 ACN:DMF at 1.2 µL/min, while the solvent used for the 100 µm imaging was 3:1 ACN:DMF at 1.4 µL/min.


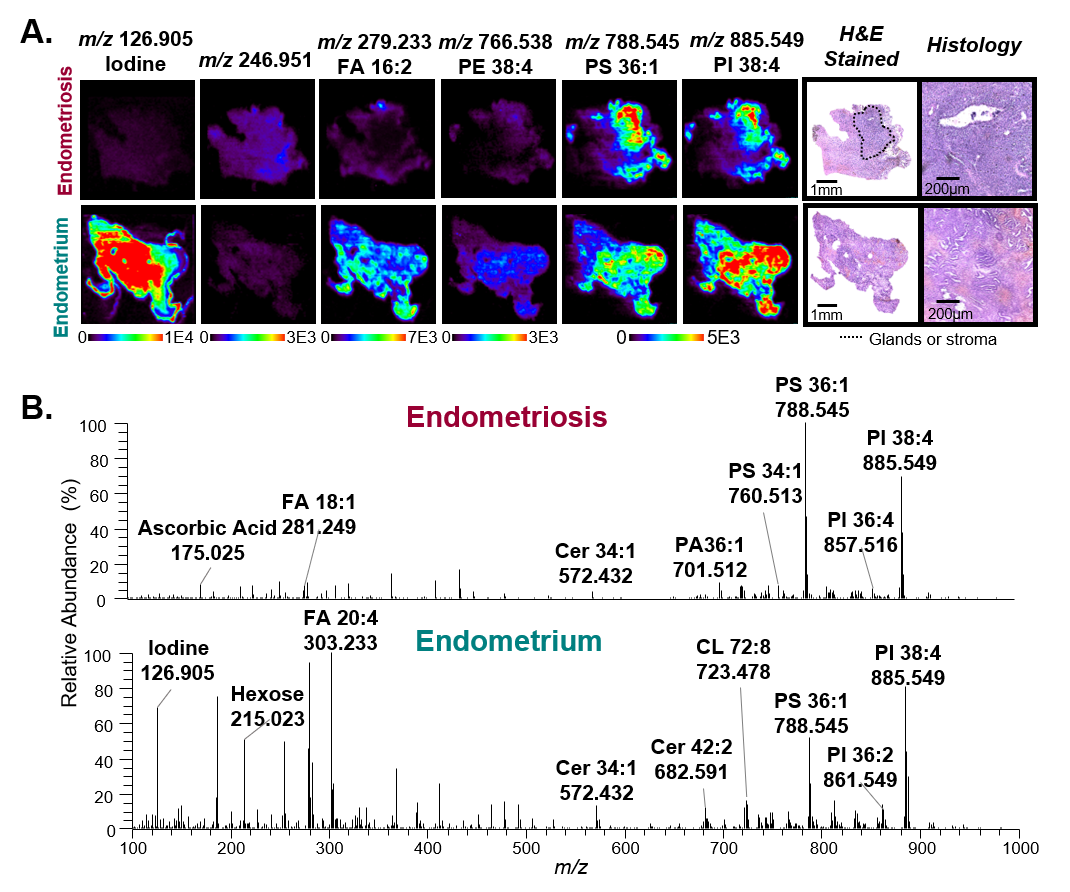


**Figure S6.** DESI-MS imaging data for endometriosis lesions and eutopic endometrium collected from the same patient. **a.** Ion images of endometriosis and endometrial tissue. For the endometriosis lesions, the regions of endometrial glands and stroma within the lesions have been outlined in black on the H&E stained optical images. **b.** Lipid and metabolite profiles for ectopic endometrial tissue collected from endometriosis lesions (Top) and eutopic endometrial tissue from inside the uterus (Bottom).


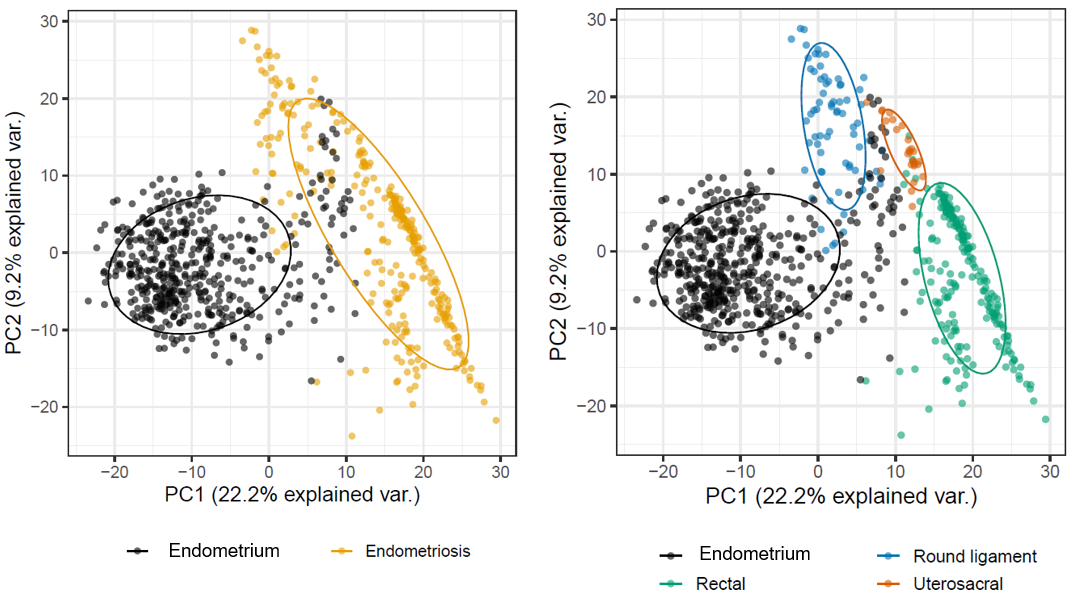


**Figure S7.** Intra-patient PCA score plots of eutopic endometrium and three endometriosis tissues collected from round ligament, rectal, uterosacral area of patient #30.


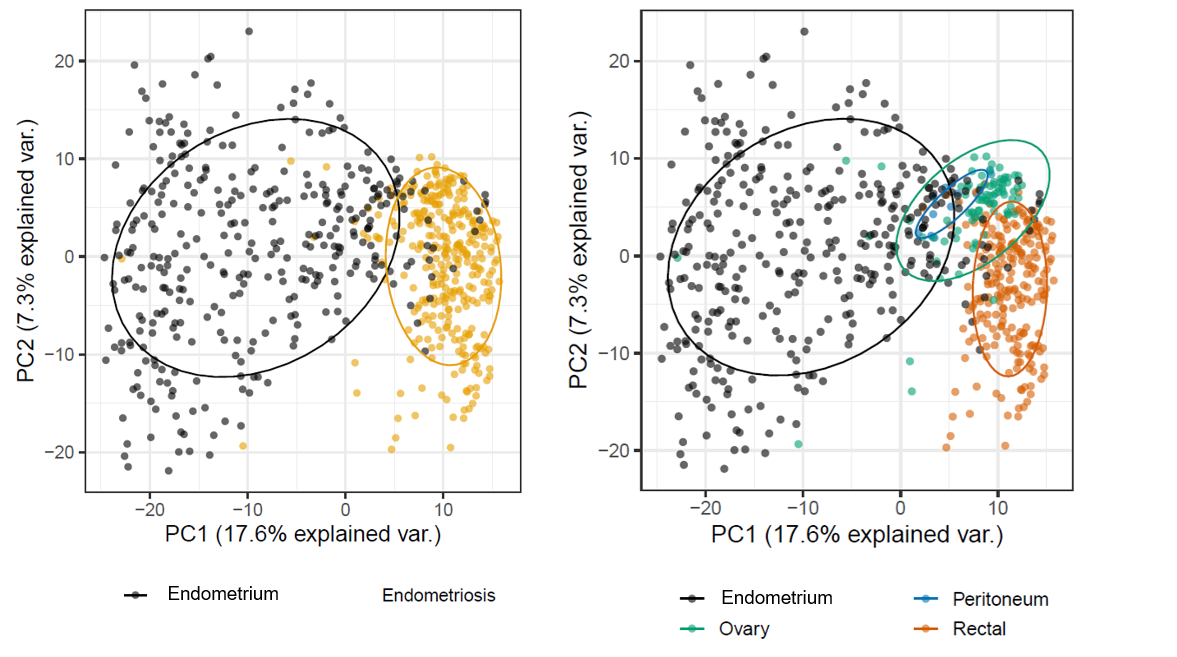


**Figure S8.** Intra-patient PCA score plots of eutopic endometrium and three endometriosis tissues collected from round ligament, rectal, uterosacral area of patient #45.


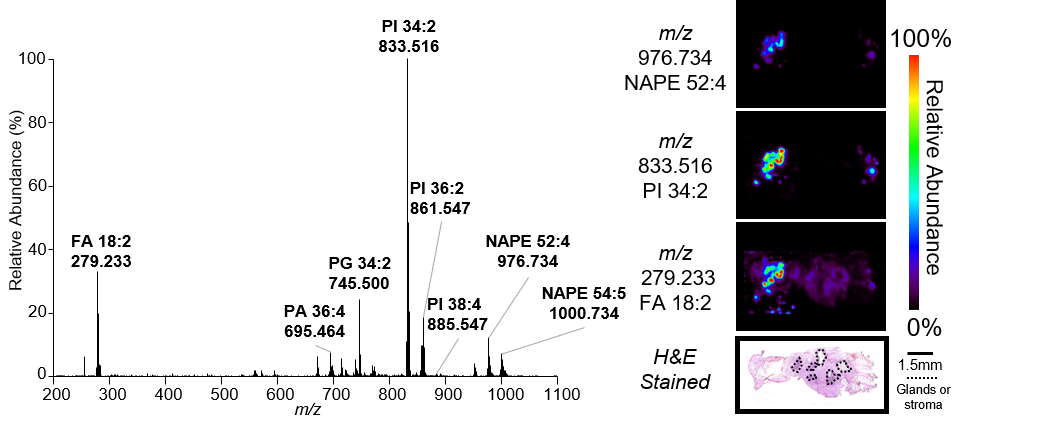


**Figure S9.** Representative DESI-MS spectrum and ion images corresponding to an endometriosis lesion tissue section presenting regions with unusual molecular profile characterized by high abundance of PI, PG, and the rare NAPE lipid species. The regions of endometrial glands and stroma within the lesions have been outlined in black on the H&E stained optical images.

**Figure S10.** Visual representation of the nonparametric bootstrapping analysis, illustrating that the majority of features within our dataset differ significantly between the eutopic and ectopic endometrial tissue using a standard null hypothesis. All features with standard deviations (error bars) falling outside of a 0 value log-fold change will be returned with a p-value of approximately zero when using a traditional null hypothesis.

Figure S11. Histogram of the *z*-values calculated from the observed log-ratio of mean ion intensities of the eutopic and ectopic endometrium tissue, divided by their standard error. The black lines overlaid on the histogram represent the estimated mixture density and the estimated null density, and selected “interesting” features are signified by green lines below the histogram.


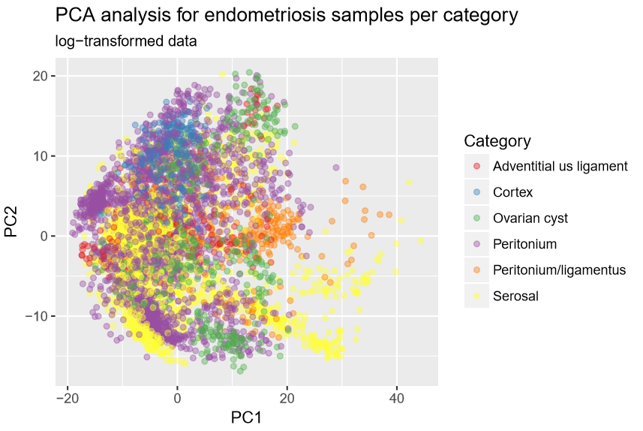


**Figure S12.** PCA score plot of ectopic endometrial tissue, grouped by region of excision, showing minimal clustering based on where the lesion was located in the patient.


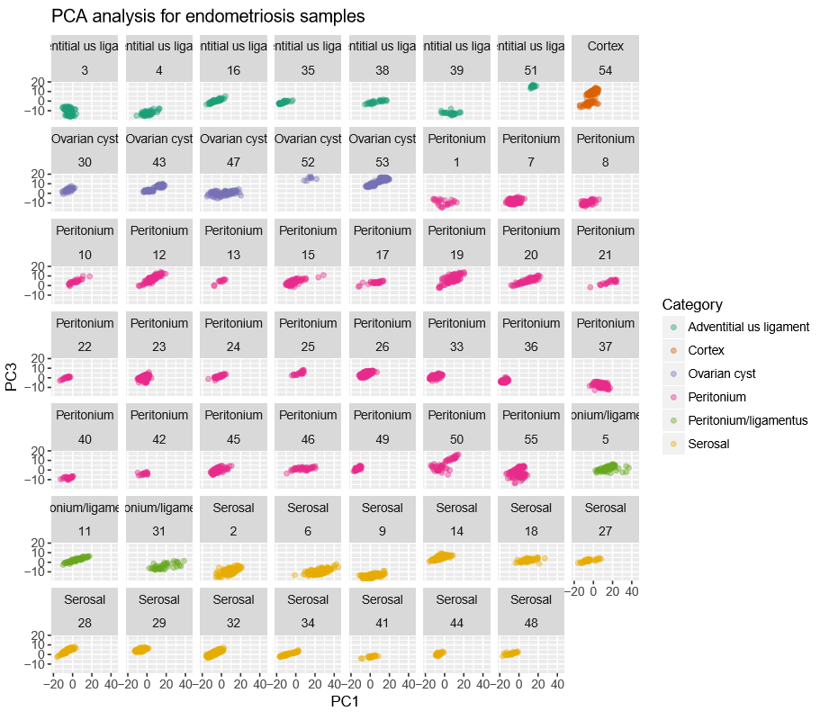


Figure S13. PCA score plots faceted by sample number and colored by location of excision.


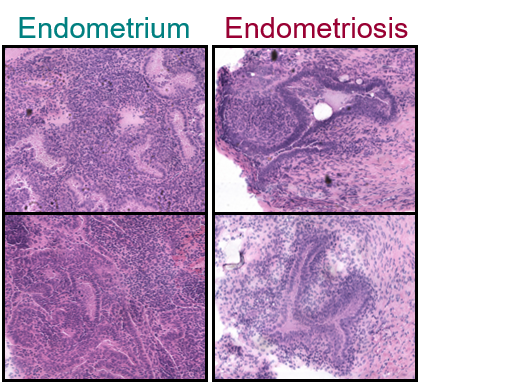


Figure S14. Comparison of eutopic endometrium (left) and endometriosis (right) glands and stroma, showing their morphological and cellular similarity.


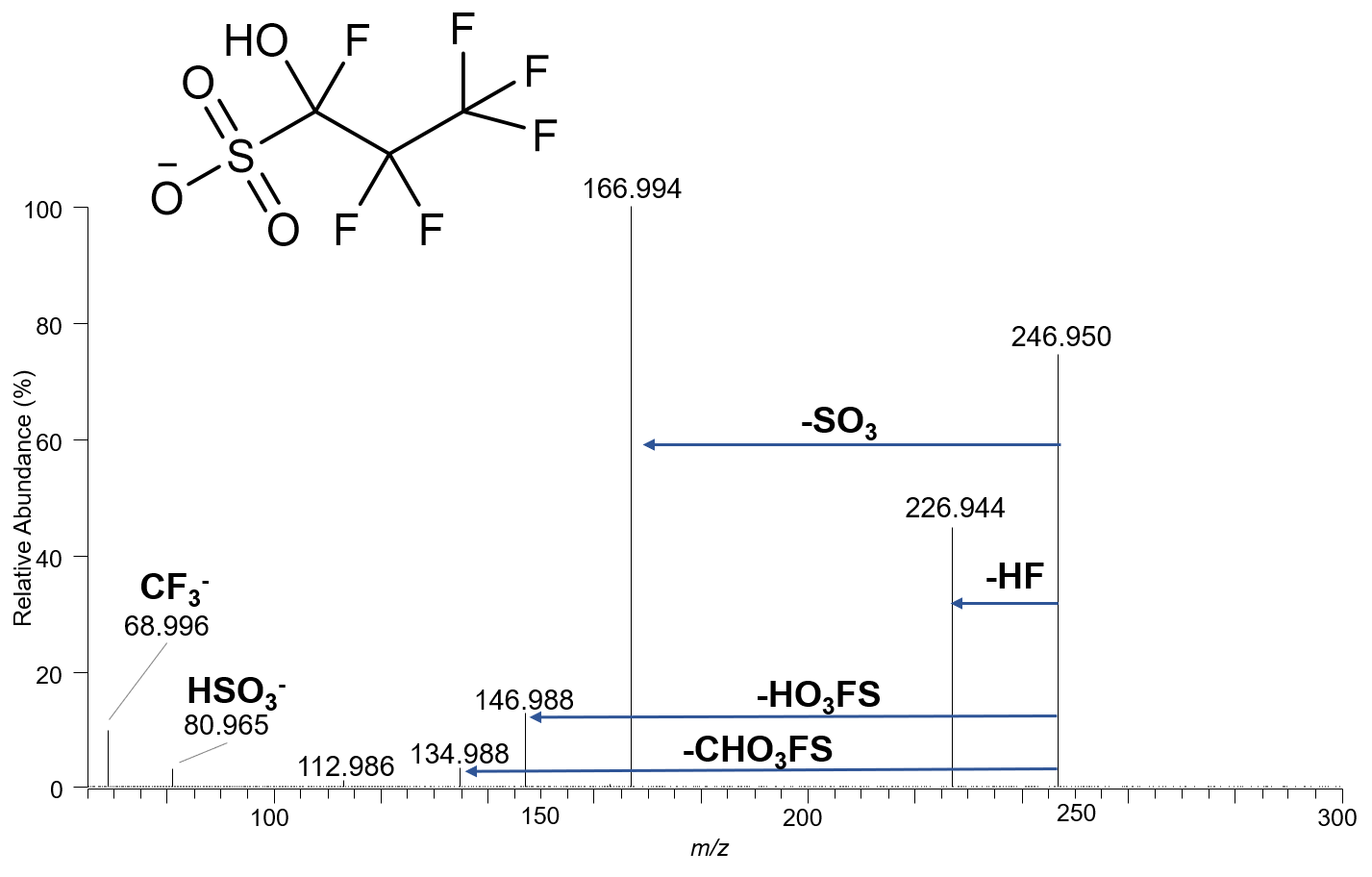


Fig. S15. Tandem MS data of *m/z* 246.951 and proposed neutral loss fragments. Due to the fluorinated nature of this compound, it is most likely a derivatization product of sevoflurane, a common surgical anesthetic.

Table S1. Summary of patient demographics for prospectively collected endometriosis samples. Note that some patients that contributed an endometriosis sample also contributed a eutopic endometrium sample, and thus the total number of patients (89) is not the sum of patients from Table S1 and S2.

| **Race** | | | | | | |
| --- | --- | --- | --- | --- | --- | --- |
| African American | Asian | East Indian | Hispanic | White | Other | Unknown |
| 8 | 1 | 1 | 9 | 53 | 1 | 3 |
| **Age** | | | | | | |
| ≤20 | 20-29 | 30-39 | 40-49 | ≥50 | Median | Mean |
| 1 | 11 | 34 | 28 | 2 | 38 | 37.8 |

Table S2. Summary of patient demographics for prospectively collected endometrium samples. Note that some patients that contributed a eutopic endometrium sample also contributed one or more endometriosis samples, and thus the total number of patients (89) is not the sum of patients from Table S1 and S2.

| **Race** | | | | | | |
| --- | --- | --- | --- | --- | --- | --- |
| African American | Asian | East Indian | Hispanic | White | Other | Unknown |
| 4 | 0 | 0 | 2 | 15 | 1 | 0 |
| **Age** | | | | | | |
| ≤20 | 20-29 | 30-39 | 40-49 | ≥50 | Median | Mean |
| 0 | 0 | 16 | 6 | 0 | 37 | 38.2 |

Table S3. Summary of menstrual cycle day information for samples used for the statistical analysis in this study. Note that the total number of patients who contributed samples used for statistics (51) is not equal to the total number of samples (98) as some patients contributed multiple endometriosis lesions on both a eutopic endometrium and one or more endometriosis lesions. Samples under the not applicable label had irregular menstrual cycles due to amenorrhea, prior hysterectomy, cycle altering birth control (intra-uterine device, Nexplanon, Depo Provera, or combined estrogen progesterone pill), endometrial ablation, continuous OCA (?), or are postmenopausal.

| Day 1-10 | Day 11-20 | Day 21-30 | Day >30 | Mean | Median | Not Applicable |
| --- | --- | --- | --- | --- | --- | --- |
| **Eutopic endometrium** | | | | | | |
| 9 | 4 | 4 | 3 | 16.4 | 12.5 | 2 |
| **Endometriosis** | | | | | | |
| 18 | 16 | 16 | 2 | 15.6 | 16 | 24 |

**Table S4.** Information regarding the number and types of samples contributed by each patient. If patients contributed ectopic endometrial samples, information about the location of excision is provided in the fourth column. Eutopic endometrial samples were all obtain from the uterus.

| **Patient number** | **Eutopic endometrial samples contributed** | **Ectopic endometrial samples contributed** | **Types of endometriosis lesions obtained** |
| --- | --- | --- | --- |
| 3 |  | 1 | Peritoneum |
| 4 |  | 1 | Serosal |
| 5 |  | 1 | Serosal |
| 12 |  | 1 | Peritoneum |
| 13 |  | 1 | Peritoneum |
| 16 |  | 2 | Peritoneum |
|  |  |  | Adventital us ligament |
| 17 |  | 3 | Peritoneum |
|  |  |  | Peritoneum |
|  |  |  | Serosal |
| 19 | 2 |  |  |
| 21 |  | 3 | Adventital us ligament |
|  |  |  | Adventital us ligament |
|  |  |  | Peritoneum |
| 23 | 1 | 1 | Adventital us ligament |
| 24 | 1 |  |  |
| 25 | 1 | 1 |  |
| 27 |  | 3 | Peritoneum and Ligamentous |
|  |  |  | Peritoneum and Ligamentous |
|  |  |  | Adventital us ligament |
| 28 | 1 |  |  |
| 30 | 1 | 4 | Serosal |
|  |  |  | Serosal |
|  |  |  | Adventital us ligament |
|  |  |  | Peritoneum and Ligamentous |
| 34 | 1 |  |  |
| 36 |  | 8 | Peritoneum |
|  |  |  | Peritoneum |
|  |  |  | Peritoneum |
|  |  |  | Serosal |
|  |  |  | Peritneum |
|  |  |  | Peritoneum |
|  |  |  | Endometrioma |
|  |  |  | Endometrioma |
| 38 | 1 | 1 | Peritoneum |

| **Patient number** | **Eutopic endometrial samples contributed** | **Ectopic endometrial samples contributed** | **Types of endometriosis lesions obtained** |
| --- | --- | --- | --- |
| 40 |  | 3 | Peritoneum |
|  |  |  | Peritoneum |
|  |  |  | Peritoneum |
| 41 | 1 |  |  |
| 42 | 1 | 2 | Peritoneum |
|  |  |  | Endometrioma |
| 45 | 1 | 3 | Peritoneum |
|  |  |  | Peritoneum |
|  |  |  | Serosal |
| 48 | 1 |  |  |
| 49 | 1 |  |  |
| 50 |  | 2 | Endometrioma |
|  |  |  | Peritoneum |
| 51 |  | 2 | Peritoneum |
|  |  |  | Peritoneum |
| 52 |  | 1 | Serosal |
| 53 | 1 |  |  |
| 54 |  | 3 | Serosal |
|  |  |  | Peritoneum |
|  |  |  | Peritoneum |
| 56 | 1 | 2 | Serosal |
|  |  |  | Peritoneum |
| 59 | 1 |  |  |
| 65 |  | 3 | Serosal |
|  |  |  | Cortex |
|  |  |  | Cortex |
| 67 |  | 1 | Peritoneum |
| 68 | 1 |  |  |
| 69 |  | 1 | Adventital us ligament |
| 71 |  | 1 | Peritoneum |
| 72 |  | 1 | Serosal |
| 74 |  | 1 | Peritoneum |
| 75 |  | 1 | Serosal |
| 76 |  | 1 | Cortex |
| 77 |  | 2 | Serosal |
|  |  |  | Peritoneum |
| 78 |  | 1 | Serosal |
| 79 | 1 |  |  |
| **Patient number** | **Eutopic endometrial samples contributed** | **Ectopic endometrial samples contributed** | **Types of endometriosis lesions obtained** |
| 82 |  | 3 | Peritoneum |
|  |  |  | Adventital us ligament |
|  |  |  | Serosal |
| 85 |  | 2 | Adventital us ligament |
|  |  |  | Serosal |
| 87 |  | 1 | Peritoneum |
| 88 |  | 1 | Peritoneum |
| 89 |  | 1 | Fallopian tube |
| 93 | 1 | 2 | Peritoneum |
|  |  |  | Adventital us ligament |
| 96 | 1 |  |  |
| 98 | 1 | 4 | Cortex |
|  |  |  | Serosal |
|  |  |  | Endometrioma |
|  |  |  | Serosal |

**Table S5.** Confusion matrix of the per-pixel and per-patient statistical results provided by the lasso model for the training, validation, and test sets of samples. Agreement is the PPA for our model for endometriosis and NPA for our model for the endometrium tissue.

|  | **Analysis** | **Pathology** | **Endometriosis** | **Endometrium** | **Agreement (%)** | **Overall Agreement (%)** |
| --- | --- | --- | --- | --- | --- | --- |
| **Training Set** | Per Pixel | Endometriosis | 3505 | 111 | 96.9 | 89.4 |
|  |  | Endometrium | 1689 | 11640 | 87.3 |  |
|  | Per Sample | Endometriosis | 43 | 1 | 97.7 | 96.6 |
|  |  | Endometrium | 1 | 14 | 93.3 |  |
| **Validation Set** | Per Pixel | Endometriosis | 1248 | 82 | 93.8 | 97.8 |
|  |  | Endometrium | 33 | 3853 | 99.2 |  |
|  | Per Sample | Endometriosis | 10 | 1 | 90.9 | 92.9 |
|  |  | Endometrium | 0 | 3 | 100.0 |  |
| **Test Set** | Per Pixel | Endometriosis | 2290 | 79 | 98.8 | 98.8 |
|  |  | Endometrium | 0 | 4263 | 100.0 |  |
|  | Per Sample | Endometriosis | 20 | 1 | 95.2 | 96.0 |
|  |  | Endometrium | 0 | 4 | 100.0 |  |

**Table S6.** Lasso weight, detected *m/z* value, proposed identification and mass error for the tissue specific features selected by the Lasso model. Features that were also selected by the nonparametric bootstrap analysis are denoted with an *. Features without a proposed identification, denoted by a – in the “Identification” column, are of unknown origin and may not be biologically relevant, but have been included for transparency.

| **Selected feature** | **Lasso Weight** | **Observed *m/z* value** | **Theoretical Mass** | **Identification** | **Mass error (ppm)** |
| --- | --- | --- | --- | --- | --- |
|  | Indicative of endometriosis | | | |  |
| 215.05* | -10.46 | 215.033 | 215.0322 | Hexose [M+Cl] | -2.3 |
| 279.23 | -4.07 | 279.233 | 279.2330 | FA 18:2 | -2.1 |
| 281.25 | -0.27 | 281.249 | 281.2486 | FA 18:1 | -1.8 |
| 788.54* | -10.19 | 788.544 | 788.5447 | PS 18:1_18:0 | -0.1 |
|  | Indicative of eutopic endometrium | | | |  |
| 126.91* | 46.34 | 126.905 | 126.9045 | Iodine [M] | -5.5 |
| 187.04* | 8.21* | 187.041 |  | --^‡^ | -- |
| 201.04* | 6.32 | 201.038 | 201.0375 | Lactate [2M+Na-2H] | -2.5 |
| 255.23 | 3.01 | 255.233 | 255.2330 | FA 16:0 [M-H] | -2.4 |
| 303.23 | 0.94 | 303.233 | 303.2330 | FA 20:4 [M-H] | -2.0 |
| 305.26 | 0.92 | 305.249 | 305.2486 | FA 20:3 [M-H] | -0.3 |
| 331.26 | 2.67 | 331.264 | 331.2643 | FA 22:4 [M-H] | -1.8 |
| 421.22 | 0.55 | 421.226 | 421.2260 | Dioctyl sulfosuccinate [M-H] | -1.9 |
| 479.36 | 3.79 | 479.356 |  | -- | -- |
| 885.56 | 0.19 | 885.549 | 885.5499 | PI 18:0_20:4 [M-H] | -1.0 |
| 887.56 | 20.34 | 887.557 | 887.5566 | PI 18:0_20:3 [M-H] | -0.5 |

‡ Indicates isotope.

**Table S7:** Z-score, detected *m/z* value, proposed identification and mass error for the tissue specific features selected by the nonparametric bootstrap method. Features that were also selected by the lasso analysis are denoted with an *. Features without a proposed identification, denoted by a – in the “Identification” column, are of unknown origin and may not be biologically relevant, but have been included for transparency.

| **Selected feature** | **z-score** | **Observed *m/z* value** | **Theoretical Mass** | **Identification** | **Mass error (ppm)** |
| --- | --- | --- | --- | --- | --- |
| Increased abundance in endometriosis | | | | | |
| 168.04 | -44.10 | 168.031 | 168.0297 | 2-furoylglycine [M-H] ^§^ | -4.8 |
| 187.01 | -53.32 | 187.007 | 187.0065 | P-Cresol Sulfate [M-H] ^§^ | -3.7 |
| 215.05* | -55.17 | 215.033 | 215.0322 | Hexose [M+Cl] | -3.7 |
| 216.03 | -38.29 | 216.036 | 216.0356 | Hexose [M+Cl] ^‡^ | -3.2 |
| 217.03 | -52.88 | 217.030 | 217.0293 | Hexose [M+Cl] ^‡^ | -2.3 |
| 254.08 | -71.44 | 254.079 | -- | -- | -- |
| 323.23 | -54.93 | 323.220 | 323.2198 | MG 14:0 [M+Na-2H]^§^ | -0.6 |
| 377.09 | -39.09 | 377.085 | 377.0851 | Disaccharide [M+Cl] | -1.3 |
| 701.52 | -72.69 | 701.512 | 701.5127 | PA 18:1_18:0 [M-H] | -0.4 |
| 702.51 | -70.29 | 702.516 | 702.5161 | PA 18:1_18:0 [M-H+1]^‡^ | -0.1 |
| 760.52 | -77.71 | 760.512 | 760.5134 | PS 16:0_18:1 [M-H] | -0.5 |
| 761.52 | -55.92 | 761.517 | 761.5168 | PS 16:0_18:1 [M-H+1]^‡^ | -0.8 |
| 771.52 | -36.15 | 771.518 | 771.5182 | PG 18:1_18:2 [M-H] | -1.8 |
| 773.53 | -36.72 | 773.533 | 773.5338 | PG 18:1_18:1 [M-H] | -3.6 |
| 788.54* | -70.31 | 788.544 | 788.5447 | PS 18:0_18:1 [M-H] | -0.1 |
| 789.55 | -67.87 | 789.547 | 789.5481 | PS 18:0_18:1 [M-H+1]^‡^ | -0.3 |
| 790.55 | -67.87 | 790.550 | 790.5514 | PS 18:0_18:1 [M-H+2]^‡^ | -0.5 |
| 791.55 | -52.22 | 791.552 | 791.5548 | PS 18:0_18:1 [M-H+3]^‡^ | -0.8 |
| 810.53 | -36.11 | 810.528 | 810.5291 | PS 18:0_20:4 [M-H] | -0.6 |
| 811.532 | -36.04 | 811.532 | 811.5326 | PS 18:0_20:4 [M-H+1]^‡^ | -0.6 |
| 844.61 | -39.92 | 844.607 | 844.6073 | PS 18:1_22:0 [M-H] | -1.2 |
| 845.61 | -39.26 | 845.611 | 845.6107 | PS 18:1_22:0 [M-H+1]^‡^ | -1.1 |
|  | Increased abundance in eutopic endometrium | | | |  |
| 124.01 | 69.70 | 124.008 | 124.0068 | Taurine [M-H] | -6.5 |
| 126.91* | 285.56 | 126.905 | 126.9045 | Iodine [M] | -5.5 |
| 151.03 | 100.60 | 151.026 | 151.0256 | Xanthine [M-H] | -5.3 |
| 186.05 | 130.60 | 186.046 | -- | -- | -- |
| 187.04* | 129.78 | 187.042 | -- | --^‡^ | -- |
| 188.06 | 114.72 | 188.062 | -- | -- | -- |
| 189.05 | 90.63 | 189.058 | -- | --^‡^ | -- |
| 201.04* | 71.70 | 201.038 | 201.0375 | Lactate [2M+Na-2H] | -2.0 |
| 307.27 | 84.25 | 307.264 | 307.2643 | FA 20:2 [M-H] | -1.6 |
| 308.26 | 69.88 | 308.268 | 308.2675 | FA 20:2 [M-H+1]^‡^ | -1.3 |
| 309.29 | 77.48 | 309.280 | 309.2799 | FA 20:1 [M-H] | -1.3 |
| 333.27 | 79.55 | 333.280 | 333.2799 | FA 22:3 [M-H] | -0.3 |
| 341.23 | 74.55 | 341.225 | 341.2245 | FA 20:3 [M+Cl] | -3.8 |
| 365.34 | 73.80 | 365.343 | 365.3420 | Tetracosanedione [M-H] | -1.6 |

‡ Indicates isotope

§ Identified with exact mass alone due to low abundance or interference with other species
